# Treating Depression in Dementia Patients: A Risk or Remedy—A Narrative Review

**DOI:** 10.3390/geriatrics9030064

**Published:** 2024-05-15

**Authors:** Sadia Sultan

**Affiliations:** Clinical Sciences Department-MBBS Program, Fakeeh College for Medical Sciences, Jeddah 21461, Saudi Arabia; sssultan@fcms.edu.sa

**Keywords:** depression, dementia, Alzheimer’s disease, antidepressants, major depressive disorder, SSRI

## Abstract

Background: The diagnosis of depression in dementia patients leads to an increase in the burden of the disease. To treat depression in this patient group, antidepressants are frequently used; however, there is not any proof of their therapeutic effectiveness, and their use may be potentially harmful. This narrative review aims to summarize the existing evidence regarding the role of antidepressants in treating depression in dementia patients. Main text: A search was conducted in the PubMed, Excerpta Medica database (EMBASE), and Cochrane databases for randomized controlled trials and meta-analyses wherein antidepressants were given to dementia sufferers to address depression. Fifteen randomized controlled trials and seven meta-analyses were identified. Most well-designed blinded placebo-controlled trials reported a lack of effectiveness of antidepressants in treating depression in dementia patients. Among the seven metanalyses, two reported good efficacy of Selective serotonin reuptake inhibitors (SSRIs). However, two major Cochrane reviews reported little or no effectiveness and increased side effects of antidepressants in dementia patients. Conclusion: There is robust evidence regarding the lack of efficacy of antidepressants in treating depression in dementia patients. However, further well-designed Randomized controlled trials (RCTs,) using scales with good validity and reliability to diagnose depression in dementia patients, sufficient sample sizes, and detailed adverse effect profiles may help determine the rationale for their use.

## 1. Introduction

In dementia patients, depression is a prevalent comorbidity. It is estimated that up to 14.8% of persons who have Alzheimer’s disease (AD) and up to 24.7% of those who have vascular dementia (VaD) have major depressive disorder (MDD), respectively [1]. Antidepressants are commonly used in patients with depression and dementia; however, there is little evidence that they are therapeutically effective [2]. They are also used to treat behavioral and psychological symptoms of dementia (BPSD), such as aggression, agitation, and apathy [3].

Evaluating the role of antidepressants in people with dementia might be difficult due to the difficulties in recognizing and tracking depressive symptoms in this patient population. Although depression and dementia are viewed as separate clinical entities, they have several characteristics in common, such as apathy, irritability, agitation, changes in sleep and appetite, attentional deficit, and an impaired working memory [4]. Another difficulty in diagnosing depression in dementia patients is their lack of ability to report symptoms of depression. However, such difficulty in reporting depressive symptoms is more likely in severely demented patients [5]. As a result, the development of depressed symptoms is frequently misunderstood as progressive cognitive deterioration, resulting in underdiagnosis. Undetected depression adds to patient disability and caregiver burden. There is a need to effectively treat depression and BPSD as they cause increased caregiver burden [6], increased cost of care [7], and poor quality of life [8]. 

To address the challenges in the diagnosis of depression in dementia patients, Olin’s provisional diagnosis criteria were proposed, which allow the diagnosis of depression with the presence of three symptoms versus the five symptoms (in the DSM-5) required for the diagnosis of MDD [9]. 

The use of tricyclic antidepressants (TCAs), such as imipramine, amitriptyline, and clomipramine, is associated with a negative impact on cognition, due to their anticholinergic side effects [10]. Other anticholinergic effects include increased intra-ocular pressure, urinary retention, dry mouth, and constipation. An increased risk of falls, due to postural hypotension caused by antiadrenergic side effects, is another important side effect noticed with TCAs [11]. Serotonin reuptake inhibitors (SSRIs) are less likely to confuse and cause falls, due to their less marked anticholinergic and antiadrenergic side effects, making these the most commonly prescribed antidepressants for the elderly [12]. Serotonin–norepinephrine reuptake inhibitors (SNRI) cause more side effects than SSRIs, and duloxetine is more associated with falls in the elderly [13]. Moreover, the American Geriatric Society recommended using SSRIs and SNRIs with caution, due to hyponatremia as a result of syndrome of inappropriate antidiuretic hormone (SIADH) [14]. Nevertheless, given the adverse effects of these medications, it is questionable if any antidepressants should be used to treat depression in dementia patients. Furthermore, there are little data to support their effectiveness in treating depression in dementia patients.

In systematic reviews and metanalyses, the usefulness of antidepressants in treating depression in dementia patients has generally been found to be poorly supported or ambiguous [15,16,17]. Despite the lack of evidence, there has been a substantial increase in antidepressant prescriptions in Canada [18] and the UK [19]. As a result of their extensive usage and the lack of conclusive evidence regarding their therapeutic efficacy and adverse effects profile in populations with dementia, it is crucial to prove their efficacy and tolerability in this condition. Therefore, this study analyses the effectiveness and tolerability of antidepressants in dementia patients, as well as the reasons why doctors continue to prescribe them.

## 2. Method

### 2.1. Type of Review

A narrative review was preferred due to the nature of the data which were available and the scope of the topic addressed; it was apt to adopt this approach rather than a systematic review. Multiple queries in a large body of varying-quality literature are not well-served by formal systematic review techniques.

### 2.2. Studies Considered for This Review

The PUBMED, EMBASE, and Cochrane databases were searched to find relevant studies. All relevant studies prescribing antidepressants for depression in dementia patients, published from 1993 to October 2023, were searched. PubMed was searched using the terms (a) ‘‘antidepressants OR imipramine OR clomipramine OR amitriptyline OR citalopram OR duloxetine OR escitalopram OR fluoxetine OR fluvoxamine OR nefazodone OR paroxetine OR sertraline OR venlafaxine OR Vortioxetine OR mirtazapine or moclobemide’’ AND (b) ‘‘depression OR major depressive disorder’’ AND (c) ‘‘Alzheimer’s OR Alzheimer’s disease OR AD OR Alzheimer’’. The results were filtered to include only RCTs. For inclusion, RCTs were required to have an antidepressant treatment arm administered to a population with dementia. Trials were excluded if the sample population was cognitively healthy or had mild cognitive impairment. All studies in languages other than English were excluded. Adults with AD, diagnosed in accordance with the DSM-V diagnostic criteria or the criteria of the National Institute of Neurological and Communicative Diseases and Stroke–Alzheimer’s Disease and Related Disorders Association (NINCDS-ADRDA), with concomitant depression diagnosed according to the DSM-5, constituted the study population. We excluded studies of patients with dementia suffering from emotional disorders or behavioral problems but falling short of a diagnosis of depression. We searched the PubMed and Cochrane Library databases for relevant systematic reviews to be included in the study.

**Figure 1:**
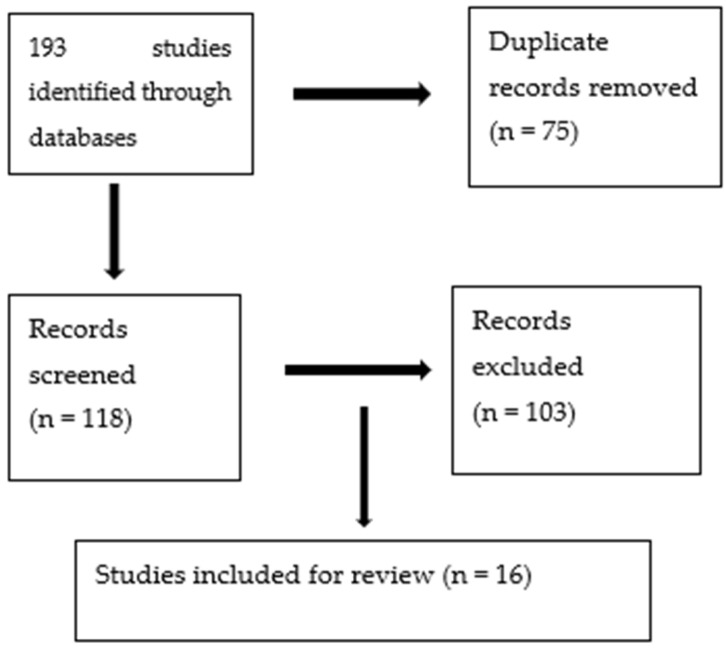
Selection process of included studies in the review.

The Cochrane risk-of-bias 2 tool for randomized controlled trials (RoB 2 tool) [20] was used to judge whether each trial was at a high, low, or unclear risk of bias. The majority of the studies included had unclear or low risks of bias. The reasons for the exclusion of studies were as follows: studies using no well-defined criteria for the diagnosis of depression and dementia, studies designed to assess the effect of antidepressants on cognition, studies designed to assess the effect of antidepressants on the behavioral and psychological symptoms of dementia but not depression, studies of antidepressant use for depression in elderly people who were not demented, studies that lacked a placebo group, and studies in which all participants were demented but comorbid depression was present in less than 50% of the sample. Trials that used stimulants or antipsychotics along with antidepressants were also excluded. Finally, a total of sixteen RCTs were included in the review Figure 1.

## 3. Results

### 3.1. Evidence of Antidepressant Effectiveness in Dementia Patients with Depression through Randomized Controlled Trials (RCTs)

The effectiveness of antidepressants in treating depression with dementia is poorly reported. Many RCTs reported a lack of antidepressant efficacy in dementia patients diagnosed with depression (Table 1). In this review, sixteen RCTs were included (Table 1). The majority of RCTs excluded severe dementia patients by including only those patients with Mini Mental Status Examination (MMSE) scores of >10. Most studies employed the Cornell Scale for Depression in Dementia (CSDD) scale as an outcome measure for depression and included a treatment duration of 12 weeks. The majority of trials found a decrease in depressive symptoms from baseline, although there was no significant change when antidepressant and placebo groups were compared [21,22,23]. Only four studies [24,25,26,27] showed significantly more improvement in the depression scores of the treatment group, compared to the placebo group, but these studies were small in size and did not use scales validated to measure depression in dementia patients, such as CSDD. Patrecca et al. [25] reported that clomipramine was significantly better at improving depression, while Takemoto et al. [27] and Lyketsos et al. [26] reported escitalopram and sertraline to be significantly better than the placebo, respectively. However, care should be taken when interpreting these studies, due to limitations on a methodological basis, such as small sample sizes and the use of varied scales to measure depression. The Depression in Alzheimer’s disease Study (DIAD) study of sertraline reported a significant improvement in depression when the antidepressant group was compared with the placebo group [26]. However, robust data from other studies [22,23,28,29,30] and DIAD-2 [23] have not reported any supremacy of sertraline in treating depression with dementia. The single largest study, which enrolled 326 patients, showed no differences in depression scores in sertraline and mirtazapine groups compared to the placebo group [30,31]. However, the Banerjee study was criticized for its methodological issues in terms of the recruitment and type of patients studied, doses used, and the limitations of the CSDD [32]. The inclusion of patients with dysthymia and mild depressive symptoms in the therapy group also raised additional concerns. Nevertheless, it is important to highlight that all groups in Banerjee’s study improved independently of the interventions, and the study’s participants were chosen from specialized old-age psychiatry services within the UK. Most old-age service centers use psychosocial interventions, which could have contributed to the ‘active placebo’ effect that was observed in all groups.

RCTs of fluoxetine [24], venlafaxine [22], and escitalopram [33] also showed no more change in depression scores for treatment groups than placebo groups. There are a few older studies which investigated the effectiveness of tricyclic antidepressants (TCAs) [25] and monoamine oxidase inhibitors (MAO-inhibitors) [24]. Clomipramine participants significantly improved in depression scores and remission rate compared to placebo participants [25]. Similarly, Roth et al. [24] found a high efficacy of moclobemide in improving depression. However, the clinical utility of TCAs and MAO-inhibitors has not been shown, due to the quality of the designs of these trials and the noticeable adverse effects of these types of antidepressants. The Serotonin or Mirtazapine for Depression In Dementia (HTA-SADD trial) found no efficacy of mirtazapine when compared to a placebo [30,31]. Additionally, there is a significant volume of negative event reporting; the HTA-SADD study found that 43%, 41%, and 26% of the participants reported adverse reactions, respectively [23,29,30]. The Study for Mirtazapine for agitated behaviors in Dementia (SYMBAD trial of mirtazapine for agitated behavior in dementia patients demonstrated no improvement in agitated behavior; rather, a higher mortality rate was observed with its use [34]. A newer SSRI, vortioxetine, has a broad range of effects on depressive symptoms, including cognitive symptoms [35,36]. An observational study by Cumbo E. et al. [37] reported improvement in depressive symptoms in those with mild AD [37,38]. However, an RCT reported no superiority of vortioxetine over a placebo in improving depression [39].

**Table 1:** Summary of RCTs depicting antidepressant efficacy in treating depression in dementia patients.

First Author/Year	Type of Participants	Treatment	Duration	Outcome Measure	Result
Petracca et al., 1996 [25]	NINCDS-ADRDA for probable AD; mean MMSE = 21.5; Hachinski ischemic score < 4; DSM-III-R MDD; HAM-D > 10.	Clomipramine 25 mg to 100 mg, *n* = 11; placebo, *n* = 10	6 weeks.	HDRS, MMSE, FIM score.	Significant improvement in depression scores in clomipramine group, no difference in MMSE scores.
Roth et al., 1996 [24]	DSM-III for dementia and depression; mean MMSE = 20.2; GDS ≥ 5; HAM-D > 14.	Moclobemide 400 mg/day or placebo, N = 694	6 weeks.	HDRS, MMSE.	Moclobemide was superior to the placebo in improving depression score (*p* = 0.001).
Petracca et al., 2001 [21]	NINCDS-ADRDA for probable dementia and DSM-IV for MDD and minor depression; HAM-D >14; Hachinski ischemic score < 4; mean MMSE = 23.2.	Fluoxetine 10–40 mg, *n* = 20; placebo, N = 21.	6 weeks.	HDRS, MMSE, FIM score.	The fluoxetine group did not differ significantly from the placebo group.
Lyketsos et al., 2003 (DIAD) [26]	NINCDS-ADRDA for probable AD; DSM-IV for major depressive episode; mean MMSE = 16.9.	Sertraline 95 mg/day, N = 24; placebo, *n* = 20.	12 weeks.	CSDD, HDRS.	Sertraline significantly improves CSDD score (*p* = 0.02) and HDRS score (*p* = 0.01), compared to placebo.
de Vasconcelos et al., 2007 [22]	DSM-IV for probable dementia and MDD; MMSE = 10–24.	Venlafaxine 75 mg/day, *n* = 11; placebo, *n* = 12.	6 weeks.	MADRS CGI.	No significant effect in MADRS CGI scores in venlafaxine group compared to placebo.
Rosenberg et al., 2010 [23]	DSM-IV for AD; mean MMSE = 20; Olin’s provisional diagnostic criteria for depression.	Sertraline 25–125 mg/day, *n* = 67; placebo, *n* = 64.	12 weeks.	CSDD.	No significant difference in CSDD scores between groups; sertraline group reported more adverse events.
Weintraub et al., 2010 [28]	DSM-IV dementia due to Alzheimer’s disease; mean MMSE = 20; Olin’s provisional diagnostic criteria for depression.	Sertraline 25–125 mg per day, *n* = 67; placebo, *n* = 64.	24 weeks.	CSDD.	No significant difference in improvement in CSDD score between groups.
Drye L.T. et al., 2011 DIAD-2 [29]	NINCDS-ADRDA for probable AD (MMSE = 10–26).	Sertraline (100 mg/day) vs. placebo, *n* = 131.	24 weeks.	mADCS-CGIC; CSDD.	No significant difference between CSDD and mADCS-CGIC scores between groups; significantly more side effects in the treatment group.
Banerjee S, et al., 2011 [30] and 2013 [31] (HTA-SADD) [32]	NINCDS-ADRDA for probable AD; mean MMSE = 18.1; Olin’s criteria for depression in AD l; CSDD > 8.	Sertraline 150 mg/day, * n * = 107; mirtazapine 45 mg/day, *n* = 108; placebo, *n* = 111.	13 weeks, 39 weeks.	CSDD.	Differences in CSDD scores at 13 weeks from an adjusted linear mixed model: mean difference (95% CI) placebo–sertraline 1.17 (−0.23 to 2.78; *p* = 0.102); placebo–mirtazapine 0.01 (−1.37 to 1.38; *p* = 0.991); and mirtazapine–sertraline 1.16 (−0.27 to 2.60; *p* = 0.112). Placebo group had fewer side effects than treatment group.
Romeo R. et al., 2013 [40]	Dementia: probable or possible AD; BPSD: depression lasting ≥4 weeks; CSDD > 8.	Sertraline 150 mg/day, *n* = 107; mirtazapine 45 mg/day, *n* = 108; placebo, *n* = 111.	13 weeks, 39 weeks.	CSDD.	Mirtazapine and sertraline were not cost-effective for treating depression in dementia (*p* > 0.05).
An H. et al., 2017 [33]	Dementia: probable or possible AD; MMSE score = 10–26; depression defined by Olin’s provisional diagnostic criteria; GDS > 5.	Escitalopram 15 mg/day, *n* = 27; placebo, *n* = 33.	12 weeks.	CSDD.	No significant change in CSDD scores between the two groups (*p* = 0.76); no difference in adverse effect between groups (*p* = 0.83).
Zuidersma M. et al., 2019 [27]	Dementia: probable or possible AD; BPSD: depression lasting ≥4 weeks; CSDD > 8.	Sertraline 150 mg/day, * n * = 107; mirtazapine 45 mg/day, *n* = 108; placebo, *n* = 111.	13 weeks, 39 weeks.	LCA of CSDD yielded the following four subgroups: 1. Severe; 2. Psychological; 3. Affective; 4. Somatic.	Symptom-based subgroup analysis revealed that mirtazapine was more effective in the psychological symptoms subgroup at 13 weeks (adjusted estimate: −2.77 [standard error (SE) 1.16; 95% confidence interval: −5.09 to −0.46]), which remained, but lost statistical significance at week 39 (adjusted estimate −2.97 [SE 1.59; 95% confidence interval: −6.15 to 0.20]).
Takemoto et al., 2020 [41]	Dementia: probable or possible AD; GDS > 5.	Sertraline 31.8 mg, *n* = 11; placebo, N = 11; escitalopram 7.3 mg, N = 13; placebo, *n* = 11.	12 weeks.	GDS.	Sertraline was not better than the placebo, but the escitalopram group showed a significant improvement in GDS score from the baseline (8.2 ± 3.5) to 3 M (5.7 ± 2.6, *p* = 0.04).
Banerjee S et al., 2021 (SYMBAD) [34]	Dementia: probable or possible AD; CMAI score of >45.	Mirtazapine 45 mg/day, *n* = 102; placebo, *n* = 102.	12 weeks.	CMAI.	No benefit of mirtazapine compared with placebo (adjusted mean difference −1.74, 95% CI −7.17 to 3.69; *p* = 0.53). Potentially higher mortality with use of mirtazapine (*p* = 0.065).
Jeong H.W. et al., 2022 [39]	AD diagnosis using NINCDS-ADRDA criteria; MMSE score = 10–26; depression defined by Olin’s provisional diagnostic criteria; GDS > 5.	Vortioxetine 20 mg/day, *n* = 49; placebo, *n* = 51.	12 weeks.	CSDD, GDS.	No benefit of vortioxetine over placebo (*p* > 0.05). Adverse events similar in both groups (*p* > 0.05).

AE, adverse effects; AD, Alzheimer’s disease; CGI, clinical global impression; CDR, Clinical Dementia Rating Scale; CMAI, Cohen-Mansfield Agitation Inventory; CSDD, Cornell Scale for Depression in Dementia; FIM, Functional Independence Measure; GDS, Geriatric Depression Scale; HDRS, Hamilton Depression Rating Scale; LCA, latent class analysis; MADRS, Montgomery–Åsberg Depression Rating Scale; mADCS-CGI, modified Alzheimer’s Disease Cooperative Study-Clinical Global Impression of Change; MD, mean difference; NaSSA, noradrenergic and specific serotonergic antidepressant; NPI-M, Neuropsychiatric Inventory—Mood; NINCDS-ADRDA, National Institute of Neurological and Communicative Diseases and Stroke–Alzheimer’s Disease and Related Disorders Association; PDRS, Psychogeriatric Depression Rating Scale—activities of daily living subscale; SMD, standardized mean difference; HTA-SADD, health technology assessment study of the use of antidepressants for depression in dementia; DIAD, depression in Alzheimer’s disease; SYMBAD, study of mirtazapine for agitated behaviors in dementia trial.

### 3.2. Evidence of Antidepressant Effectiveness in Dementia Patients with Depression through Meta-Analysis

Based on data from seven meta-analyses, the effectiveness of antidepressants was found to be poor and ambiguous (Table 2). A meta-analysis by Sephery et al. [42] concluded that antidepressants were ineffective. Other metanalyses have likewise reported scant evidence about the effectiveness of antidepressants in treating depression in dementia patients [42,43]. 

Two Cochrane reviews of antidepressants for treating depression in dementia [17,44] identified poor evidence regarding antidepressant use in treating depression in dementia patients. The first Cochrane review identified four studies with a fair amount of homogeneity which were then entered into a meta-analysis [17]. This review demonstrated weak evidence of antidepressant efficacy, while some positive effects of antidepressants were seen in DIAD-1 [26]. However, a more recent Cochrane review found that there was no role of antidepressants in treating depression in dementia patients [45]. This study included eight studies that were sufficiently homogenous to enter into a meta-analysis, with a total of 1592 subjects [45]. In this study, tricyclic antidepressants (TCAs), venlafaxine, mirtazapine, and selective serotonin reuptake inhibitors (SSRIs) were all independently examined in subgroup analyses, but no difference was found between treatment groups and placebo groups. This study was meticulously executed in terms of maintaining the homogeneity of the studies included. Most included studies used CSDD scores as an outcome measure, which is a specific scale designed to diagnose depression in dementia patients [12]. Moreover, most studies included in the meta-analysis were randomized double-blind placebo-controlled trials. Additionally, this study performed a subgroup analysis of studies, grouped together based on scales used to assess depression and the duration of treatment [45]. There was also adherence to the study inclusion criteria, which states that all subjects included in the review must satisfy established criteria for both depression and dementia, which helped, to some extent, to ensure homogeneity. Despite this, the chance of underdiagnosis of depression was high in this patient group. The diagnosis of depression is difficult due to the overlapping symptoms of depression and dementia, such as anhedonia, poor concentration, and apathy. Moreover, not all studies included in this review measured depression with CSDD, which is a validated tool measure depression in dementia patients. Some other scales of depression, such as HDRS and MADRS, are intended to measure depression in younger patient groups. The use of such scales may underestimate depression in older patients. Even instruments like GDS that are made explicitly for the older population might not accurately measure depression in dementia, due to the overlapping symptoms [46]. In these patients, determining therapeutic response and remission might be challenging. Heterogeneity in the reporting of adverse effects made it difficult to analyze the adverse events data in this review.

Contrary to the results of previous meta-analyses, Thompson and colleagues concluded that antidepressants were more efficacious than placebos. Thompson and colleagues [44] included five studies, four of which were included in the Cochrane review by Bains [17]. The fifth study [47] was excluded from the Cochrane review due to the fact that it included only 10% diagnosed cases of MDD, and the fact that the validity of the diagnosis of depression was questionable, as it relied on proxy measures such as the analysis of facial expression. Thompson et al.’s [47] study did not look at specific symptoms, but rather recorded treatment dropouts for all reasons and due to adverse events. From this comparatively broader approach, they discovered no notable differences, while Dudas [45] reported significantly more adverse events with the use of antidepressants.

Similarly, Zhang J. [48] discovered that antidepressant therapy, in particular sertraline, paroxetine, and escitalopram, significantly reduced depression symptoms in AD when compared to placebo treatment. The evidence that emerged from this investigation was rather heterogeneous and included some low-quality studies. The included studies, especially those which reported greater efficacy of SSRIs, had various limitations (Table 3). This meta-analysis included studies with different designs, unlike the Cochrane review [45], which only included randomized double-blind placebo-controlled trials. Additionally, it did not conduct subgroup analysis based on the type of scale used to measure depression. Many of the studies were small in size and used measures that are not validated to diagnose depression in dementia patients.

**Table 2:** Overview of systematic reviews with meta-analysis depicting the efficacy of antidepressants in the treatment of depression in dementia patients.

First Author/Year	Studies Included	Intervention (Drug vs. Placebo)	Mean Duration	Outcome Measure	Result
Bains et al. (Cochrane review), 2002 [17]	Seven RCTs; only four were entered into meta-analysis; N = 137.	Imipramine (Reifler, 1989); clomipramine (Petracca, 1996); fluoxetine (Petracca, 2001); Sertraline (DIADS, 2003).	6–12 weeks.	Change in HDRS and CSDD.	Inconclusive. Out of all studies, only Lyketsos et al. [26] showed significant improvement in depression with sertraline. Adverse effects were significantly less frequent in the placebo group.
Thompson et al., 2007 [44]	Five RCTs; N = 165.	Imipramine (Reifler, 1989); clomipramine (Petracca, 1996); sertraline (Magai, 2001); fluoxetine (Petracca, 2001); sertraline (DIADS, 2003).	6–12 weeks.	Response (↓ ≥50%); remission HDRS ≤ 7.	AD was superior to placebo for both treatment response (odds ratio [OR] 2.32; 95% confidence interval [CI], 1.04 to 5.16) and remission of depression (OR 2.75; 95% CI, 1.13 to 6.65). There were no significant differences between the two groups for change in cognition (weighted mean difference −0.71, 95% CI, −3.20 to 1.79), overall dropouts (OR 0.70; 95% CI, 0.29 to 1.66), or dropout due to AEs (OR 1.41; 95% CI 0.36 to 5.54).
Nelson et al. (2011) [16]	Seven RCTs; N = 330.	Imipramine (Reifler, 1989); clomipramine (Petracca, 1996); sertraline (Magai, 2001); fluoxetine (Petracca, 2001); sertraline (DIADS, 2003); venlafaxine (de Vas, 2007); sertraline (DIADS-2, 2010).	6–12 Weeks.	Response (↓ ≥50%); remission (HDRS ≤ 7, CSDD ≤ 6, MADRS ≤ 10).	AD was NOT superior to placebo for both treatment response (odds ratio OR = 2.12 (95% confidence interval (CI) 5 0.95–4.70) and remission of depression (OR 1.97 (95% CI 5 0.85–4.55). No increases in discontinuation rate and adverse event frequency were observed in the treatment group.
Sepehry et al., 2012 [42]	12 RCTs, 5 included in meta-analysis; *n* = 598.		12 weeks.	Change in HDRS and CSDD.	No significant effects of SSRIs in two depression nested analyses, including CSDD and HDRS (*p* > 0.05).
Ortego et al., 2017 [43]	Seven RCTs; *n* = 311.	Sertraline and mirtazapine (Banerjee, 2011); three studies of sertraline (Lyketsos, 2003, Rosenberg, 2010, and Magai et al., 2000); clomipramine (Petracca, 1996); fluoxetine (Petracca, 2001); imipramine (Reifler, 1989).	13 weeks, 39 weeks.	Change in HDRS, CSDD, and MADRS.	No significant drug/placebo differences for depressive symptoms (SMD −0.13; 95% CI −0.49 to 0.24).
Dudas et al. (Cochrane review), 2018 [45]	10 RCTs; *n* = 15,928; eight studies entered into meta-analysis.	Escitalopram (An, 2017); sertraline and mirtazapine (Banerjee, 2011); venlafaxine (de Vasconcelos, 2007); sertraline (Rosenberg, 2010 and Weintraub, 2010); maprotiline (Fuchs, 1993); moclobemide (Roth, 1996); sertraline (Lyketsos, 2003); clomipramine (Petracca, 1996); fluoxetine (Petracca, 2001).	13 weeks 39 weeks	Response (↓ ≥50% on HDRS, mADCS-CGI rating of 2 or <); remission (HDRS ≤ 7, CSDD ≤ 6, MADRS ≤ 10)	No difference in scores on depression symptom rating scales between the antidepressant and placebo treated groups at 6 to 13 weeks (SMD −0.10, 95% confidence interval (CI) −0.26 to 0.06 No difference at 24 to 39 weeks (MD = 0.59, 95% CI −1.12 to 2.3); subgroup analyses of various types of AD did not indicate a difference in efficacy; treatment group was significantly more likely to experience AEs; no differences in measures of cognitive function or activities of daily living and quality of life between antidepressant and placebo groups were found; AEs occurred more in participants given antidepressants compared to those on placebos.
Zhang, J. et al., 2023 (HTA-SADD) [48]	15 RCTs; one prospective cohort; *n* = 510.	Three studies of escitalopram; one study of citalopram; three studies of fluoxetine; one study of paroxetine; seven studies of sertraline.	12 weeks.	Change in CSDD, HDRS, MADRS, and GDS.	Escitalopram, paroxetine, and sertraline significantly alleviated depressive symptoms in AD patients (0.813 SMD, 95% CI, 0.207 to 1.419, *p* = 0.009, 1.244 SMD, 95% CI, 0.939 to 1.548, *p* < 0.000, and 0.818 SMD, 95% CI, 0.274 to 1.362, *p* < 0.000).

AE, adverse effects; AD, Alzheimer’s disease; CDR, Clinical Dementia Rating Scale; CMAI, Cohen-Mansfield Agitation Inventory; CSDD, Cornell Scale for Depression in Dementia; GDS, Geriatric Depression Scale; HDRS, Hamilton Depression Rating Scale; HTA-SADD, health technology assessment study of the use of antidepressants for depression in dementia; LCA, latent class analysis; MADRS, Montgomery–Åsberg Depression Rating Scale; MD, mean difference; mADCS-CGI, modified Alzheimer’s Disease Cooperative Study-Clinical Global Impression of Change; NaSSA, noradrenergic and specific serotonergic antidepressant; NINCDS-ADRDA, National Institute of Neurological and Communicative Diseases and Stroke–Alzheimer’s Disease and Related Disorders Association; SMD, standardized mean difference.

## 4. Discussion

This narrative review, which included 16 RCTs and six meta-analyses, suggests a lack of evidence for antidepressant use in treating depression in patients with dementia. As a matter of fact, antidepressant usage in dementia is not only useless but also perhaps dangerous, because it is reported to increase the risk of falls, hospitalization, and mortality [55,56]. From the above review, it is evident that many studies did not use well-defined and relevant sample populations and validated measures to assess depression in dementia. Many of them lacked methodological precision in terms of blinding and allocation concealment. Future research should focus on recruiting a large number of patients so that the findings can be generalizable to the population, the meticulous use of blinding strategies and allocation concealment, greater specificity in diagnosing depression in dementia patients, the assessment of side effects, and the comparison of risk versus benefit of use. Such trials are considerably more likely to help produce clinically relevant data that can guide physicians in treating such patients.

It is significant to note that this review has a number of restrictions. First, even though every attempt was made to find relevant literature by using broad search terms and reviewing citations, some studies were probably overlooked. Because this is a single-author review, the inclusion and exclusion of studies was based on the consensus of a single evaluator and there is a higher risk of missing papers. Grey literature was not included. The RCTs included in our study were also part of systematic reviews which were included, which might have introduced bias in interpretation. Effect size has not been calculated because this review is not a meta-analysis.

Even though there is little and conflicting research on the effectiveness of antidepressants as depression treatment in Alzheimer’s disease, they are frequently given. Studies suggest that there was an increase in prescription rates from 26% in 2010 to 31% in 2014 [54,57,58]. The dearth of practice recommendations that offer clinicians considerable direction on how to treat this important clinical issue may be the cause of such prescriptions that lack supporting data. According to the American Psychiatric Association, antidepressants can be prescribed to treat clinically significant persistent low mood, despite acknowledging that the data on antidepressants’ efficacy in individuals with dementia and depression are conflicting [59]. Most clinical practice guidelines recommend the use of antidepressants, with some variations. According to the World Health Organization’s (WHO) recommendations, psychiatric medications are justified in situations of moderate-to-severe depression [60]; nevertheless, National Institute Of Excellence NICE emphasizes the necessity of evaluating the risk–benefit ratio on an individual basis [61]. Other organizations, such as the Fourth Canadian Consensus Conference on the Diagnosis and Treatment of Dementia (CCCDTD4), support medication therapy if non-pharmacological treatment is ineffective [62]. According to the Delphi consensus, which gathered opinions from top neurodegenerative disease physicians, a unanimous agreement was achieved on the effectiveness of antidepressants; however, improvement was lessened when compared to that in patients with cognitively healthy brains. The consensus reported no change in dose and treatment period for dementia patients and reported that cholinesterase inhibitors work synergistically with antidepressants and show improvement in depressive symptoms [63].

While the precise etiology of depression in dementia is yet unknown, the most likely explanations are as follows: (1) vascular disease; (2) alterations in glucocorticoid steroids and hippocampal atrophy; (3) increased deposition of β-amyloid plaques; (4) inflammatory changes; and (5) deficits of nerve growth factors or neurotrophins [64]. Another cause of depression in dementia seems to be the degeneration of brain areas involved in mood regulation, motivation, and memory, such as the striatum, entorhinal cortex, hippocampus, pre-frontal cortex, and amygdala [65]. Therefore, expecting an improvement with antidepressants is a debatable question. Most antidepressants work either by blocking the reuptake pump or via acting on receptors of serotoninergic, noradrenergic, and dopaminergic neurons [66]. Thus, the degeneration of these neurons is therefore probably affecting the pharmacodynamics and therapeutic efficacy of antidepressants. Antidepressants show clinically significant benefits in treating clinical symptoms and avoiding relapse in depression without dementia [67]. However, expecting the same benefit in a patient population with dementia with progressive brain degeneration is debatable. Moreover, the nature of the association between depression and dementia still remains unclear [65], further increasing the ambiguity about the use of antidepressants in dementia patients.

To address the heterogeneity of depression in dementia, Fariha et al. [68] divided this clinical entity into three different groups. In Group 1, depression is a reaction to the diagnosis of dementia, which can be improved with psychological support and counseling services, rather than antidepressants [69]. This could be the explanation for the placebo response noted in previous studies. Group 2 have symptoms of depression but the actual cause is the degeneration of the locus coeruleus, substantia nigra [70], and central superior nucleus [70,71]. The loss of these nuclei is linked to serotonin and noradrenaline deficiencies, both of which are linked to mood [70]. This degeneration may explain the lack of response to antidepressants. Group 3 have a past history of depression with dementia or develop a new episode of MDD with dementia. Although the antidepressant response in these cases may be comparable to that of depression without dementia, the response may even be less, due to the neurodegenerative and neurochemical changes seen in dementia [69,70]. Awareness of such heterogeneity and the cause of depression in this patient group may help to develop patient-tailored treatments.

## 5. Conclusions

Although there is robust evidence regarding the lack of efficacy of antidepressants in treating depression in dementia patients, further well-designed RCTs using scales validated in older people with depression and dementia, sufficient sample sizes, and detailed adverse effect profiles are needed to support their use. In conclusion, the current practice of prescribing antidepressants is less driven by scientific evidence and more by a benevolent desire to control behavior without the hazards of other drugs, such as antipsychotics.

## Figures and Tables

**Table 3:** Summary of limitations of included studies in meta-analysis by Zhang et al., 2023. [48].

Study	Outcome	Limitations
Nyth et al., 1990 [49]	Citalopram showed a significant improvement in emotional bluntness, anxiety, and depressed mood.	Combined double-blind and open techniques; looked at the effect of citalopram on emotional disturbance in patients with various subtypes of dementia; patients were not diagnosed with depression according to any recognized criteria.
Tarango et al., 1997 [50]	Fluoxetine and amitriptyline were equally effective but fluoxetine was better tolerated.	Randomized trial of amitriptyline vs. fluoxetine, for patients with probable AD and major depressive disorder, but it was not placebo-controlled.
Katona et al., 1998 [51]	Paroxetine and imipramine were both effective in the treatment of dAD.	Not a placebo-controlled trial; imipramine was compared with paroxetine in cognitively impaired and depressed patients.
Lyketsos et al., 2003 [25]	Sertraline was superior to the placebo.	Recruitment of patients from specialty clinic; strict criteria applied for diagnosis of dementia and MDD, so these findings may not apply for mild mood disturbances and other types of dementias.
Rao et al., 2006 [52]	Escitalopram was efficacious and safe for the treatment of dAD.	Open labelled study design; small size; no blinding or allocation concealment.
Mowla et al., 2007 [53]	Concomitant use of SSRI and Ach-I reported improved global functioning.	The patients were not diagnosed with depression; outcome was improvement in daily activity.
Mokhber et al., 2014 [54]	Sertraline better for treating depression than venlafaxine and desipramine.	No placebo group; small size; use of HDRS.
Takemoto et al., 2020 [41]	Serotonin had better efficacy than escitalopram.	Randomized single-blind prospective observational study; no placebo; small study; used GDS to measure depression.

AD, Alzheimer’s disease; dAD, depression in Alzheimer’s disease; GDS, Geriatric Depression Scale; HDRS, Hamilton Depression Rating Scale; Ach-I, acetylcholinesterase inhibitor; SSRI, serotonin reuptake inhibitor.

## Data Availability

The authors confirm that the data supporting the findings of this study are available within the article and the Supplementary Materials.

## Supplementary Materials

The following supporting information can be downloaded at: https://www.mdpi.com/article/10.3390/geriatrics9030064/s1, Table S1. revised Cochrane Risk of bias 2 tool for randomized controlled trials (RoB 2 tool).

## Abbreviations

AChEIs	acetylcholinesterase inhibitors
AD	Alzheimer’s disease
CMAI	Cohen-Mansfield Agitation Inventory
CDR	Clinical Dementia Rating Scale
CSDD	Cornell Scale for Depression in Dementia
DIAD	depression in Alzheimer’s disease
DSM-IVTR	Diagnostic and Statistical Manual of Mental Disorders (fourth edition, text revision)
GDS	Geriatric Depression Scale
HDRS	Hamilton Depression Rating Scale
HTA-SADD	health technology assessment study of the use of antidepressants for depression in dementia
mADCS-CGI	modified Alzheimer’s Disease Cooperative Study-Clinical Global Impression of Change
MAOIs	monoamine oxidase inhibitors
MMSE	Mini-Mental State Examination
MADRS	Montgomery–Åsberg Depression Rating Scale
NPI-M	Neuropsychiatric Inventory-Mood
NaSSA	noradrenergic and specific serotonergic antidepressant
NINCDS-ADRDA	National Institute of Neurological and Communicative Diseases and Stroke–Alzheimer’s Disease and Related Disorders Association
PDRS	Psychogeriatric Depression Rating Scale—activities of daily living subscale
SSRI	selective serotonin reuptake inhibitor
TCAs	tricyclic antidepressants
SMD	standardized mean difference
SYMBAD	study of mirtazapine for agitated behaviors in dementia trial
