# Supplementary material for: Treating Depression in Dementia Patients: A Risk or Remedy—A Narrative Review
Source: Geriatrics (Basel). 2024 May 15;9(3):64. doi: 10.3390/geriatrics9030064 (PMC11130822; doi:10.3390/geriatrics9030064)
Supplement: Supplementary file 1 [file geriatrics-09-00064-s001.zip › geriatrics-2910357-supplementary.pdf]

geriatrics

Table S1. revised Cochrane Risk of bias 2 tool for randomized controlled trials (RoB 2 tool).

	Bias arising from the randomization process	Bias due to deviations from intended interventions	Bias due to missing outcome data	Bias in the measurement of outcome	Bias in selection of the reported result	Overall bias
Petracca; etal 1996	high	low	unclear	low	low	Unclear risk
Roth, et al ,1996[	unclear	unclear	unclear	low	high	Unclear risk
Petracca; etal 2001	unclear	unclear	unclear	low	low	Unclear risk
Lyketsos; et al 2003	low	low	low	low	low	low
deVasconcelos et al,2007	unclear	unclear	low	unclear	unclear	Unclear risk
Rosenberg;et al, 2010	high	unclear	unclear	unclear	high	Unclear risk
Weintraub;et a;.2010	high	unclear	high	unclear	unclear	Unclear risk
Drye LT et al, 2011 DIAD-2	unclear	high	unclear	high	unclear	Unclear risk
Banerjee S; et al, 2011	low	low	low	low	low	low
Banerjee S; et al, 2013	low	low	low	low	low	low
Romeo R; et al.2013	unclear	low	low	low	low	low
An H;etal ,2017[	unclear	unclear	unclear	high	high	Unclear risk
Zuidersma M;et al, 2019	unclear	low	unclear	low	low	low
Takemoto et al., 2020	unclear	low	unclear	low	low	low
Banerjee S;etal 2021	low	low	low	low	low	low
Jeong HW;et al 2022	unclear	low	unclear	low	low	low
